# Microencapsulation of Olive Oil by Dehydration of Emulsion: Effects of the Emulsion Formulation and Dehydration Process

**DOI:** 10.3390/bioengineering10060657

**Published:** 2023-05-28

**Authors:** Donia Chaabane, Iman Mirmazloum, Asma Yakdhane, Emna Ayari, Krisztina Albert, Gyula Vatai, Márta Ladányi, András Koris, Arijit Nath

**Affiliations:** 1 Department of Food Process Engineering, Institute of Food Science and Technology, Hungarian University of Agriculture and Life Sciences, Ménesi Str. 44, HU-1118 Budapest, Hungary; donia.chaaben@gmail.com (D.C.); asma-yk@hotmail.com (A.Y.); albert.krisztina.zita@uni-mate.hu (K.A.); vatai.gyula@uni-mate.hu (G.V.); or arijit.nath@uni-mate.hu (A.N.); 2 Department of Plant Physiology and Plant Ecology, Institute of Agronomy, Hungarian University of Agriculture and Life Sciences, Ménesi Str. 44, HU-1118 Budapest, Hungary; mirmazloum.seyediman@uni-mate.hu; 3 Department of Refrigeration and Livestock Technology, Institute of Food Science and Technology, Hungarian University of Agriculture and Life Sciences, Ménesi Str. 44, HU-1118 Budapest, Hungary; ayari.mna@gmail.com; 4 Department of Applied Statistics, Institute of Mathematics and Basic Science, Hungarian University of Agriculture and Life Sciences, Villányi út 29-43, HU-1118 Budapest, Hungary; ladanyi.marta@uni-mate.hu

**Keywords:** microencapsulation of olive oil, maltodextrin, whey protein isolate, emulsion, spray-drying, freeze-drying

## Abstract

Microencapsulation of extra virgin olive oil has been taken into consideration. Initially, emulsions were prepared using extra virgin olive oil and aqueous solutions of different proportions of maltodextrin (MD) having dextrose equivalent (DE) 19 and whey protein isolates (WPI), such as 100% MD, 100% WPI, 25% MD + 75% WPI, 50% MD + 50% WPI and 75% MD + 25% WPI. Subsequently, emulsions were used for dehydration by either spray-drying (SD) or freeze-drying (FD) to produce olive oil microcapsules. Emulsion stability, viscosity and droplet size influenced the characteristics of the microcapsules. The highest encapsulation efficiency was achieved using 50% MD + 50% WPI in the emulsions with subsequent SD. The moisture content of the microcapsules increased with increasing proportions of MD. The size of the microcapsules increased with increasing proportions of WPI. The bulk density and tapped density were reduced with higher proportions of MD in the microcapsules. Furthermore, microcapsules with a higher proportion of MD exhibited poor flowability and high cohesiveness. Microcapsules from the higher proportion MD emulsions, followed by SD were spherical with a smooth surface; however, microcapsules with dent structures were produced from 100% WPI in the emulsions with subsequent SD. Microcapsules, produced from emulsions with a higher proportion of WPI, followed by FD were flat flakes and had irregular surfaces.

## 1. Introduction

The wide range of beneficial outcomes has catapulted olive oil to the forefront of the functional food sector. Olive oil is enriched with monounsaturated fatty acids (ω-6 and ω-3 fatty acids), antioxidant phenolics and flavonoids, secoiridoids, vitamin E and vitamin K. However, olive oil is widely produced in the Mediterranean region; presently, it has received popularity around the globe for its fine taste, aroma and nutritional properties [1]. Furthermore, the development of olive oil for biopharmaceuticals, to reduce the risk of several chronic and acute metabolic disorders, is noteworthy [2,3]. Presently, olive oil has ushered health-conscious consumers into a new fraternity of sophisticated consumers, conscious of regular wellness. According to the recommendation of the World Health Organization (WHO) the ratio ω-6/ω-3 in olive oil is highly satisfactory [2]. Therefore, the application of olive oil for the formulation of a ketogenic diet apart from a regular diet has been taken into consideration [4].

However, extra virgin olive oil is enriched with antioxidants; often due to the oxidation of fatty acids during processing, distribution and handling, organoleptic and nutritional properties of the olive oil are deteriorated [5]. Oxidation of fatty acids produces hydroperoxides, carboxylic acids, aldehydes, ketones, short chain alkenes and alkanes [6]. Thus, the microencapsulation of olive oil has been considered as an attractive approach to maintain the quality and biological activities of olive oil against oxidation [7,8].

Microencapsulation is a frontier technology, used to protect bioactive compounds within the matrix, and controls the release of bioactive compounds to the environment [9]. In this process, bioactive compounds, in liquid or solid forms, are surrounded by a thin film coating known as a wall material or matrix. It has been reported that the different characteristics of microcapsules, such as moisture content, bulk density, tapped density, flowability, cohesiveness, size and shape depend on the characteristics of the wall material and technology of the microcapsule preparation. Wall materials could act as a barrier and may protect the encapsulated bioactive compounds against oxygen, water, light and contact with other ingredients. Furthermore, the characteristics of wall materials influence the controlled release of encapsulated bioactive compounds to the environment [10]. Criteria to select wall material for the microencapsulation of bioactive compounds are (a) solubility in water, (b) a tendency to form a fine and dense network during processing, (c) a high glass transition temperature (*T*_g_) to avoid the stickiness of microcapsules and (d) resistance to the leakage of bioactive compounds during processing [11]. Unfortunately, there is no material that can meet all the mentioned criteria [8,12]. For example, maltodextrin (MD) and whey protein isolates (WPI) are taken into consideration. MD, a known polysaccharide, is hydrophobic and has a high *T*_g_. Therefore, a glassy thin film is formed more easily, and the adhesive strength and stability of the microcapsules are increased by MD. WPI is used an emulsifier due to its amphiphilic nature and has film-forming properties due to its low *T*_g_. Several disadvantages have been reported when they were used alone to develop a matrix for the microencapsulation of vegetable oils. MD is not recommended to be used alone as a matrix because of its poor emulsifying properties [8]. The application of WPI alone is not appreciable because it has high water activity with low *T*_g_ [13]. Commonly, spray-drying (SD) and freeze-drying (FD) are used in food and feed industries for the microencapsulation of bioactive ingredients [14]; however, spray granulation and liposome entrapment are also used for the microencapsulation of bioactive compounds [15].

To reduce the limitation of wall materials for microencapsulation of bioactive compounds, combinations of different wall materials have been used often. It has been proven by several investigations that the use of a combination of proteins and saccharides provide an appreciable encapsulation efficiency (EE) for olive oil microcapsules. For example, olive oil emulsions were prepared by a combination of MD and WPI using a rotor-stator homogenizer, followed by the dehydration of the emulsion using SD. It was noted that the EE of olive oil microcapsules was higher when MD and WPI were in the emulsion formulation than when only WPI was used [8]. In other investigations, emulsions have been prepared using different combinations, such as (a) gelatin, gum arabic and MD, (b) sodium caseinate and lactose, (c) sodium caseinate and MD and (d) MD and modified starch. Subsequently, the dehydration of the emulsions has been performed by SD to prepare olive oil microcapsule. The EEs of microcapsules from the emulsions were 42.35%, 52.98%, 38.60% and 45.23%, respectively [12]. Furthermore, it has been reported that the types of protein influence the microencapsulation of olive oil. Different biopolymers, such as soy protein isolate (SPI), pea protein isolate (PPI), defatted milk powder and octenylsuccinic anhydride-modified starch (OSA) were used for the microencapsulation of CoQ10 in olive oil. MD was used with the mentioned biopolymers at a 2:1 weight ratio to improve the microencapsulation. It was found that SPI with MD and OSA with MD provided similar EE of 86.5–88.5%, which is significantly higher than the other two preparations [16].

The objective of this investigation was to understand the effects of the wall material (matrix) and process of dehydration for the microencapsulation of extra virgin olive oil (unrefined and unblended olive oil). In our investigation, commercially available, extra virgin olive oil produced by cold pressing of the olive fruit was used. MD and WPI were used as matrices. In the first stage, emulsions were prepared with aqueous solutions of MD and WPI with olive oil. Different proportions of MD and WPI were used for the preparation of the emulsions. A fixed amount of Tween 20 was used as an emulsifier. Emulsion was characterized by stability, viscosity, size of emulsion droplets and their distribution. In a later exercise, the emulsion was used for the preparation of olive oil microcapsules by dehydration. Two different dehydration technologies, such as SD and FD were adopted. Microcapsules were characterized by the EE, particle size and their distribution, moisture content, bulk density, tapped density, flowability, cohesiveness and surface morphology.

## 2. Materials and Methods

### 2.1. Materials

Extra virgin olive oil (unrefined and unblended olive oil) was purchased from a local supermarket in Budapest, Hungary. Olive oil was stored in a glass bottle in the dark at room temperature (RT) (~25 °C). MD, having dextrose equivalent (DE) of 19, and WPI were purchased from the Buda Family Kft., Budapest, Austria. Tween 20, solvents and standards for chromatographic purposes were purchased from Sigma Aldrich, St. Louis, MO, USA. All other chemicals, with analytic grade, were purchased from Fluka, Darmstadt, Germany. Milli-Q ultrapure deionized (DI) water (18.2 MΩ·cm) was obtained from a Milli-Q Synergy/Elix water purification system (Merck-Millipore, Molsheim, France) and used in all experiments.

### 2.2. Emulsion Preparation

MD and WPI were dissolved in DI water by gentle shaking to avoid the formation of foam at RT. Aqueous solutions were prepared with different proportions (weight basis) of MD and WPI, such as 100% MD (100 MD), 100% WPI (100 WPI), 25% MD and 75% WPI (25 MD−75 WPI), 50% MD and 50% WPI (50 MD−50 WPI) and 75% MD and 25% WPI (75 MD−25 WPI). Tween 20 was used as an emulsifier. Subsequently, olive oil was added to the mentioned mixtures, and the ratio of olive oil to MD, WPI or MD−WPI was 1:2.3 (weight basis) [12]. To prepare the emulsions, a laboratory-scale rotor-stator homogenizer (DLAB D-160, Scilogex, Rocky Hill, CT, USA) was used. The homogenizer was operated at 10,000 rpm for 5 min at RT [8,17]. After the preparation of the emulsions, they were subjected to either FD or SD to prepare olive oil microcapsules. The compositions of emulsions are described in Table 1.

### 2.3. Dehydration by SD

A laboratory-scale spray dryer (LabPlant SD-05, Keison, Chelmsford, UK) was used for dehydration of emulsions. The pressure of the compressed air was adjusted to 36 × 10^4^ Pa. Temperatures of inlet and outlet airs were maintained at 190 ± 2 °C and 100 ± 4 °C, respectively. The airflow rate was adjusted to 74 m^3^·h^−1^. The nozzle diameter and emulsion flow rate were 0.5 mm and 15 mL·min^−1^, respectively. The microcapsules were collected from the collecting glass chamber and stored in a sample container under dark conditions at a temperature of 4 °C until they were analyzed [12].

### 2.4. Dehydration by FD

Emulsions were kept in the freezer at a temperature of −40 °C for 24 h and then used for FD (Scanvac coolsafe 110–4 apparatus, Labogene, Lillerød, Denmark). FD of the emulsion was performed at a temperature of −109 °C and a vacuum pressure ~12 Pa for 24 h. After FD, samples were manually ground to obtain a fine powder [18].

### 2.5. Analytical Methods

#### 2.5.1. Charecteristics of Extra Virgin Olive Oil

##### Extinction Coefficients

Extra virgin olive oil in cyclohexane (1% (*w*/*v*)) was used for determining extinction coefficients, such as K_232_ and K_270_ on a UV spectrophotometer (Thermo Scientific™, Waltham, MA, USA). Absorbances of solutions were measured at wavelengths of 232 nm and 270 nm [19].

##### Peroxide Value (PV)

To understand the PV of extra virgin olive oil, 1.5 g of olive oil was dissolved in 25 mL of a solvent mixture (chloroform and acetic acid (2:3, *v*/*v*)) in a volumetric flask. Then 1 mL of a saturated potassium iodide solution was added, and the mixture was shaken for 1 min and left in the dark for 5 min at RT. Subsequently, 75 mL of DI water was added. The titration of the liberated iodine was performed with a 0.01 N sodium thiosulphate solution and 1% aqueous starch solution as an indicator. The PV is expressed in terms of milliequivalents of active oxygen per kilogram of olive oil (meq O_2_·kg of olive oil^−1^).

(1) $$PV=\frac{V\times T\times 1000}{m}$$

where *V* is the amount of standardized sodium thiosulphate solution (in mL) used for the test, *T* is the exact molarity of the sodium thiosulfate solution and m is the weight in g of the test portion [20].

##### Acid Value

The acid value (titratable acidity) of the experimental olive oil was measured to understand the amount of free fatty acids in the extra virgin olive oil. Olive oil in isopropanol (10% (*w*/*v*)) was neutralized by a 0.1 N potassium hydroxide solution. Here, oleic acid (C18:1) was used as a basis because it is the predominant fatty acid in extra virgin olive oil [21].

##### Total Phenolic Content

The content of total phenolics in extra virgin olive oil was determined by the Folin–Ciocalteu method [22]. Briefly, 5 g of experimental olive oil was mixed with 5 mL of methanol-water (80:20 (*v*/*v*)) and shaken for 30 min. Subsequently, the mixture was used for centrifugation at a rotor speed of 1700× *g* for 5 min in a laboratory cold centrifuge (HERMLE Labortechnik, Wehingen, Germany). A total of 1 mL of extract was mixed with 1.5 mL of sodium carbonate (20% (*w*/*v*)) and 0.25 mL of Folin–Ciocalteu reagent in a 10 mL volumetric flask. The final volume was achieved using DI water. Samples were stored for 90 min under dark conditions at RT. The spectrophotometric analysis was performed at a wavelength (λ) of 725 nm with gallic acid (GA) as the standard. A UV-Vis spectrophotometer (Thermo Scientific™, Waltham, MA, USA) was used for the colorimetric analysis.

##### Tocopherol Composition

Sample preparation for the analysis of tocopherols in extra virgin olive oil was performed prior to injection to the LiChrospher Si60 column (inner diameter 25 cm, length 4.6 mm and particle size 5 µm) (Merck, Darmstadt, Germany), fitted in an Agilent 1200 high performance liquid chromatography (HPLC) system equipped with a fluorescent detector (Agilent Technologies, Santa Clara, CA, USA). The sample preparation method is herein: 0.15 g of olive oil was diluted in 10 mL of n-hexane followed by centrifugation with a rotor speed of 25,000× *g* for 10 min in a laboratory cold centrifuge (HERMLE Labortechnik, Wehingen, Germany). The supernatant was transferred to a chromatographic vial and 20 µL of the sample was injected into the HPLC system. Chromatographic separation was performed by the isocratic mixture of isopropanol:hexane 0.5:99.5 (*v*/*v*) mobile phase, operated with a 0.7 mL·min^−1^ flow rate. The fluorescence detector was set to excitation and emission wavelengths of 296 nm and 330 nm, respectively. Peaks were identified on the basis of the retention times of the standards α-, β-, γ- and δ-tocopherol separately and their concentrations were calculated using respective external calibration curves [23].

##### Sterol Composition

For the analysis of sterols in extra virgin olive oil, 0.2 g of olive oil was dissolved in 1 mL of n-hexane and 0.2 mL of a 5α-cholestane solution (0.4 mg·g^−1^) as an internal standard. Subsequently, the sample mixture was saponified by adding 0.5 mL of an ethanolic potassium hydroxide solution (20 mL of ethanol and 50% (*w*/*v*) potassium hydroxide) at RT for 2 h. The unsaponifiable fraction was extracted with diethyl ether. Subsequently, the extract was transferred into a sample vial and dried under a nitrogen stream. The extract was further dissolved in 1.5 mL of hexane and transferred into a sample vial followed by drying under a nitrogen stream. In the next step, the residue was re-dissolved in 100 μL of pyridine and 100 μL N,O-bis (trimethylsilyl) trifluoroacetamide (BSTFA) with 1% trimethylchlorosilane (TMCS) and heated in temperature 60 °C for 1 h for sample derivatization purpose. Subsequently, 1 mL of heptane was added to the dried sample, and the obtained mixture was analyzed using a ZB-5ms capillary column (inner diameter 30 m, length 0.25 mm and film thickness 0.25 μm) (Phenomenex, Torrance, CA, USA) in a gas chromatography-mass spectrometry (GC-MS) system (QP2010 PLUS, Shimadzu, Kyoto, Japan). Separation of sterols was performed with helium as a carrier gas with the flow rate at 1 mL·min^−1^. A 1 µL of sample was injected in splitless mode and the injector temperature was 230 °C. The column temperature of the GC was programmed as follows: initial temperature 50 °C for 2 min with a subsequent increase to 230 °C at the rate of 15 °C·min^−1^, and then to 310 °C at a rate of 3 °C·min^−1^ with a 10 min hold. The inlet pressure was maintained at 28.5 psi. The interface temperature of the GC-MS was 240 °C. The temperature of the ion source and electron energy were 220 °C and 70 eV, respectively. The total ion current (TIC) mode was used for quantification (100–600 *m*/*z* range). The identification of the peaks was based on a comparison of their mass spectra with standard compounds and their quantifications were carried out using cholestanol as an internal standard [24].

##### Fatty Acid Composition

The fatty acids composition of extra virgin olive oil was determined from their methyl ester forms by an Agilent 6850 gas chromatography system (Santa Clara, CA, USA) using the capillary column DB-23 (inner diameter 0.25 mm, length 60 m and film thickness 0.25 µm) and a hydrogen flame ionization detector (FID). Preparation of the fatty acid methyl esters (FAME) was performed according to Mousavi et al., 2021 [25]. Briefly, 0.1 g of experimental olive oil was dissolved in 2 mL of n-heptane. In the reaction, 0.2 mL of 2 N methanolic potassium hydroxide was used as a catalyst. The reaction mixture was centrifuged with a rotor speed of 1700× *g* for 15 min in a cold laboratory centrifuge (HERMLE Labortechnik, Wehingen, Germany) and the supernatant was used for analysis. The temperatures of injector and detector were set to 230 °C and 280 °C, respectively. The injection volume was 1 µL and helium was employed as carrier gas with a flow rate of 1 mL·min^−1^ and the split ratio was 1:50. The oven temperature was programmed as follows: 140 °C for 2 min, increased to 240 °C at a rate of 4 °C·min^−1^ which was held for 15 min, and then 240 °C for 42 min [26]. Fatty acids in extra virgin olive oil were identified by the comparing retention times of the methylated fatty acid standards according to Ansorena and Echarte, 2012 [27].

#### 2.5.2. Charecteristics of Emulsion

##### Emulsion Stability

Stabilities of the emulsions were measured by the percentage separation of liquids. It is considered that a stable emulsion has a lower phase separation. Immediately after emulsion preparation, 25 mL of the emulsion was transferred to a graduated cylinder and sealed with paraffin paper. Cylinders were stored at RT for 24 h and the volume of the upper phase was measured. The following correlation was used for this purpose.

(2) $$\%\;\mathrm{Separation}=\frac{H_{1}}{H_{0}}\times 100$$

where *H*_0_ and *H*_1_ represent the emulsion initial height and upper phase height, respectively [28].

##### Emulsion Viscosity

The viscosities of the emulsions were measured at RT through the determination of the steady shear flow curve (shear stress × shear rate) using a controlled-stress modular, compact rheometer (MCR92, Anton Paar, Graz, Austria). The measurement system contains a cylindrical measuring body (CC 27) with a diameter of 27 mm and the measuring head ST 24 2V-2V-2D. The viscosities of sample solutions were tested with a shear rate of 10 s^−1^ to 300 s^−1^ at a temperature of 20 °C. Rheograms were analyzed according to empirical models and viscosity was calculated from the relationship between shear stress and shear rate [29].

##### Emulsion Droplet Size and Distribution (Span)

The droplet sizes of emulsions were measured immediately after emulsion preparation. Size distributions of droplets in the emulsions were determined using a laser particle size analyzer instrument (Bettersize ST, Bettersize Instruments Ltd., Dandong, China). A small amount of emulsion was suspended in water under agitation and the droplet size distribution was monitored during each measurement until successive readings became constant. Diameters of droplets were expressed as the *D*_43_ value (DeBroukere mean). The size distributions of the droplets were determined by the span value, calculated from the following equation.

(3) $$\mathrm{Span}=\frac{D_{90}-D_{10}}{D_{50}}\times 100$$

where *D*_10_, *D*_50_ and *D*_90_ correspond to 10, 50 and 90 volume % of microcapsule diameters, respectively, on the relative cumulative dimensional distribution curve [29].

#### 2.5.3. Charecteristics of Olive Oil Microcapsules

##### Encapsulation Efficiency of Microcapsules

Microcapsules (1.5 g) and hexane (15 mL) were mixed in a screw-cap glass tube and were shaken for 2 min at RT. The solvent mixture was filtered through a Whatman filter paper 1 and the permeate from the filter paper was used for measuring the surface oil of microcapsules. The retentate from the filter paper was rinsed 3 times with 20 mL of hexane and used for measuring the total oil. Subsequently, the solvent was evaporated at a temperature of 60 °C. The weight difference between the initial clean flask and after extraction of oil residue was noted [30]. Total oil was assumed to be equal to the initial oil, since preliminary tests revealed that all the initial oil was retained due to the non-volatile nature of olive oil [31]. EE was calculated based on the following equation.

(4) $$\%\;\mathrm{EE}=\frac{T_{0}-S_{0}}{T_{0}}\times 100$$

where *T*_0_ and *S*_0_ are the total oil and surface oil in g, respectively [30].

##### Moisture Content of Microcapsule

Moisture content of olive oil microcapsule was measured gravimetrically using a moisture analyzer (KERN MLS; KERN & SOHN GmbH, Balingen, Germany). A stable heating temperature of 70 °C was maintained until a constant weight was reached [8].

##### Size of Microcapsule

The diameters of the microcapsules (*D*_43_), prepared by SD technology, were measured using a laser particle size analyzer instrument (Bettersize ST, Bettersize Instruments Ltd., China). A small amount of microcapsules was suspended in anhydrous ethanol and the size distribution was monitored during each measurement until successive readings became constant. The value of *D*_43_ was used to determine the diameter of the microcapsules. The size distribution of microcapsules was determined by the span value, as described previously in [29]. Values of *D*_43_ of the microcapsules prepared by the FD technology were not measured because they were ground manually.

##### Bulk Density and Tapped Density

A total of 2.5 g of the microcapsules were poured into a 10 mL graduated measuring cylinder and the bulk density was calculated according to the following equation [32].

(5) $$\mathrm{Bulk}\;\mathrm{density}\;\left(g\cdot {\mathrm{cm}}^{-3}\right)=\frac{\mathrm{Sample}\;\mathrm{weight}\;\mathrm{in}\;g}{\mathrm{Sample}\;\mathrm{volume}\;\mathrm{in}\;\mathrm{mL}}$$

Similarly, 2.5 g of microcapsules were transferred into a 10 mL graduated measuring cylinder, and were gently dropped 100 times onto a rubber mat from a height of 15 cm to determine the tapped volume. Tapped density was calculated according to the following equation [32].

(6) $$\mathrm{Tapped}\;\mathrm{density}\;\left(g\cdot {\mathrm{cm}}^{-3}\right)=\frac{\mathrm{Sample}\;\mathrm{weight}\;\mathrm{in}\;g}{\mathrm{Total}\;\mathrm{volume}\;\mathrm{in}\;\mathrm{mL}}$$

Values of bulk density and tapped density of microcapsules prepared by FD technology were not measured because after FD, flakes were ground manually. Therefore, the sample volume was inconsistent in every experiment.

##### Flowability and Cohesiveness

The flowability and cohesiveness of the microcapsules were determined based on the Carr index and the Hausner ratio. For that purpose, bulk density and tapped density of microcapsules were used. The following corelations were adopted for those purposes [32].

(7) $$\mathrm{Carr}\;\mathrm{index}\;\left(\%\right)=\frac{\mathrm{Tapped}\;\mathrm{density}-\mathrm{Bulk}\;\mathrm{density}}{\mathrm{Tapped}\;\mathrm{density}}\times 100$$

(8) $$\mathrm{Hausner}\;\mathrm{ratio}\;(-)=\frac{\mathrm{Tapped}\;\mathrm{density}}{\mathrm{Bulk}\;\mathrm{density}}$$

Carr index and Hausner ratio of microcapsules prepared by FD technology were not measured because the results of bulk density and tapped density of microcapsules produced by FD were not available. Reason was mentioned earlier.

##### Morphology of Microcapsule

A field emission scanning electron microscope (FESEM) (JSM 5500 LV, Jeol Ltd., Tokyo, Japan) was used in the experiment. Olive oil microcapsules were coated by a combination of gold and platinum (60:40) for 10 min with 10 mA plasma current. Coated samples were placed in SEM for analyzing its surface morphology. In FESEM, backscattered secondary electron (BSE) flow was used [33].

### 2.6. Statistical Analysis

All experiments were performed in triplicate and the mean values with standard deviations (S.D.) were calculated using an IBM SPSS (v27, Armok, NY, USA: IBM Corp., 2020). Significant differences between different groups in emulsions were determined by the one-way multivariate analysis of variance (MANOVA) method in cases of variables, such as stability in terms of % separation (-), *D*_43_ (µm), span (-) and viscosity (mPa.s). Two-way MANOVA models were used for microcapsules in cases of EE (%) and moisture content (%). Furthermore, the one-way multivariate analysis of variance (MANOVA) method was performed in the cases of variables *D*_43_ (µm), span (-), bulk density (g·mL^−1^), tapped density (g·mL^−1^), Carr index (%) and Hausner ratio (-) for microcapsules. Having significant MANOVA results, univariate ANOVA models were run with Bonferroni’s correction in all cases. The differences were considered significant when *p* < 0.05. Stability data was previously transformed by the square-root function to ensure the normality of the residuals that was tested by Kolmogorov-Smirnov test (*p* > 0.05). The homogeneity of variances was checked by the Levene’s test (*p* > 0.05) and slight heteroscedasticity of EE was detected. Finally, whether homoscedasticity was violated or not in any case, we separated significant groups by the Games-Howell’s test or the Tukey’s post hoc test, respectively.

## 3. Results and Discussion

### 3.1. Characterization of Olive Oil

The characteristics of extra virgin olive oil depend on the genetic variety of olive fruit, the degree of olive ripening, crop season, geographical area and climate [34,35]. In the present experiment, 6–12 months old extra virgin olive oil (according to the mentioned manufacturing time on the bottle), procured from a local supermarket in Budapest, Hungary was used. Extinction coefficients, such as K_232_ and K_270_ were found to be 1.45 ± 0.001 and 0.17 ± 0.001, respectively. The peroxide value of our experimental olive oil was 8.02 ± 0.74 meq O_2_·kg of olive oil^−1^. It may be realized that studied extra virgin olive oil exhibited the values of some quality indices (K_232_ ≤ 2.5; K_270_ ≤ 0.22 and peroxide value ≤20 meq O_2_·kg of olive oil^−1^) within the limits, established by the EU regulation for extra virgin olive oil [36]. Total phenolic content of the experimental olive oils was 480 ± 02 mg GA·kg of olive oil^−1^. Different types of tocopherols, such as α-, β- and γ-tocopherol were 270 ± 0.4 mg·kg of olive oil^−1^, 2.5 ± 0.01 mg·kg of olive oil^−1^ and 0.4 ± 0.001 mg·kg of olive oil^−1^, respectively; however, δ-tocopherol was not found in our experimental olive oil. The acid value of extra virgin olive oil was 0.35% of C18:1. The fatty acid profiles in our experimental olive oil are indicated in Table 2. It is noted that oleic acid C18:1, palmitic acid and linoleic acid are abundant in our experimental olive oil. The composition of fatty acids in that olive oil was within the guidelines of the International Olive Council (IOC), 2008 [37]. Furthermore, the composition of sterol in extra virgin olive oil is presented in Table 2. It is noted that β-sitosterol, Δ-5-avenasterol, campesterol, stigmasterol and clerosterol are the abundant phytosterols in our experimental olive oil.

### 3.2. Characterization of Emulsion

#### 3.2.1. Emulsion Stability

An emulsion is characterized by its stability, viscosity and droplet size. The percentage of phase separation in emulsions represents the stability of emulsion [38]. In Table 3, it is shown that the emulsion is more stable when the proportion of MD is higher in the emulsion formulation. MD contains linear amylose regions that have an internal helical structure with a hydrophobic nature and branched amylopectin region. Highly aqueous soluble MD may produce a stable emulsion because MD may form a local hydrophobic region within the emulsion and facilitates hydrophobic interactions with oil droplets [39]. On the other hand, it is noted that WPI offers poor stability (higher phase separation) for the emulsion. In the dairy industry, WPI is produced by the dewatering of liquid whey and subsequent dehydration by SD. Heating causes the unfolding of protein structures and subsequently creates aggregation of the protein structure [40]. High-speed homogenization may cause the unfolding of protein molecules. Unfolded protein molecules at the oil–water interface may enhance protein–protein interactions and promote agglomeration. Therefore, as the droplet size of the emulsion is increased, the stability of the emulsion is reduced in the presence of WPI in emulsion formulation [28].

#### 3.2.2. Emulsion Droplet Size and Distribution (Span)

Droplet diameters (*D*_43_) of the emulsions are presented in Table 3. It is noted that the droplet size of the emulsions was reduced with an increasing proportion of MD in the emulsion. MD is highly soluble in water and the hydrophobic areas of MD with DE 19 are more exposed to interactions with the oil droplets [41]. Therefore, coalescence among droplets in the emulsion is minimized. The droplet size of the 100 WPI emulsion is smaller than the 25 MD−75 WPI, 50 MD−50 WPI and 75 MD−25 WPI. It may be justified by the fact that the movements of droplets inside the emulsion are difficult when the emulsion has a higher viscosity and droplet coalescence in the emulsion is restricted [38].

The span value of the emulsion is considered an indicator of the dispersity of droplets within the emulsion. In Table 3, span values of the emulsions are presented. Lower span values signifie the monodisperse nature of droplets in the emulsion [8]. It is noted that emulsions are more monodispersed due to the presence of MD in the emulsion formulation. On the other hand, the concentration of WPI in the emulsions leads to aggregation of droplets and influences the dispersity of emulsion.

#### 3.2.3. Emulsion Viscosity

**Figure 1:**
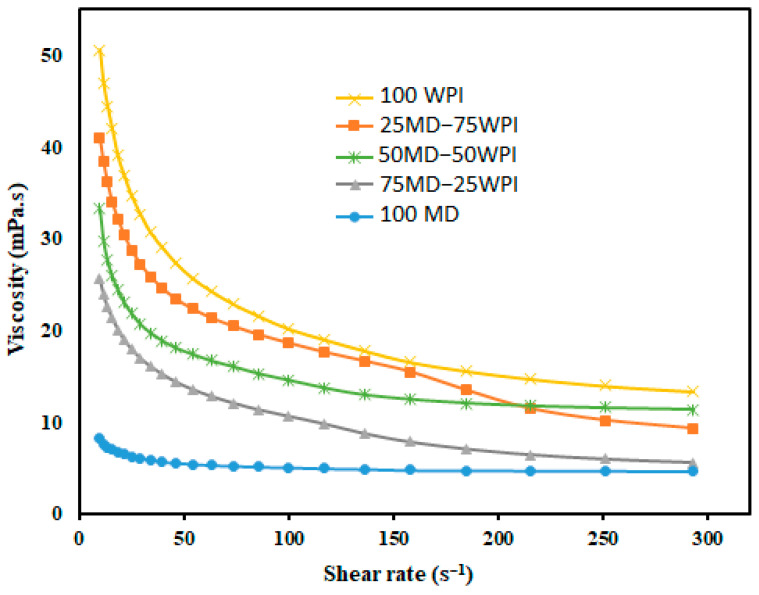
Viscosity of emulsions (mPa.s) prepared by olive oil with the different proportions of MD and WPI. MD: Maltodextrin, WPI: Whey protein isolate.

The viscosities of emulsions prepared by olive oil and the different proportions of MD and WPI in water are shown in Figure 1. However, the 100 MD emulsion is characterized as a Newtonian fluid, whereas others are characterized as non-Newtonian fluids. The viscosity was reduced by the high proportion of MD in the emulsions and the lowest viscosity was noted for the 100 MD emulsion (Table 3). It has been proven that MD with a higher DE value, such as 19, contains mostly low molecular weight saccharides, which produces emulsions with lower viscosities [42]. The viscosity of the 100 WPI emulsion is considerably higher compared to other emulsions. It is worth noting that viscosity was improved when the proportion of WPI was increased in the emulsion formulation. It has been reported that heat-induced whey protein aggregates lead to higher intrinsic viscosity than native whey protein in solution [43].

### 3.3. Microcapsule Characterization

#### 3.3.1. Encapsulation Efficiency (EE)

The EEs for microcapsules produced by the different proportions of MD and WPI and different dehydration processes (SD and FD) are presented in Table 4. It is noted that microcapsules from the 100 WPI emulsion have higher EE than microcapsules from the 100 MD emulsion. In an investigation, it has been proven that the encapsulation of fatty acids by WPI was appreciable due to the higher proportion of proteins in WPI, which promotes hydrophilic–hydrophobic interactions. Lack of hydrophobic regions in MD compared with WPI may provide the lower EE of fatty acids [44]. The EE of microcapsules from the 100 WPI emulsion was 49.67 ± 1.86, and it was increased significantly due to the incorporation of MD in emulsion formulation, such as 50 MD−50 WPI. It has been reported that the combination of WPI and MD with a higher DE provided better stability to the oil-in-water emulsion under mild acidic (pH 6), neutral (pH~7) and alkaline (pH 8–9) conditions [45]. A stable emulsion may produce a microcapsule with appreciable EE. The appreciable emulsifying property of protein due to the amphiphilic nature, together with the hydrophobic nature and film-forming property of MD offer higher EE for the microcapsules [46]. According to Table 4, the EEs of microcapsules from the 75 MD−25 WPI emulsion and 100 MD emulsion are significantly lower than microcapsules from the 50 MD−50 WPI emulsion. It might be the retro-degradation or aggregation of the hydrophobic regions in the helical structure of MD due to the increase of MD in the emulsion formulation, which can influence the loss of oil content during SD [39]. In contrast, the EEs of microcapsules produced by the FD are significantly lower than SD (Table 4); however, it is expected that the quality of olive oil could be maintained after FD of the emulsion. This result could be attributed to the destabilization of the emulsion during freezing for 24 h. FD significantly affects the microstructure and integrity of matrices of oil droplets. Therefore, a matrix with a porous and irregular surface is produced, which promotes oil leakage and lower EE [47]. Similar results were published by other investigators [14,48,49].

#### 3.3.2. Moisture Content

Moisture content of microcapsules is associated with water activity, prevention of lipid oxidation in capsules from oxidative agents during storage [50], shelf life and microbial spoilage [51]. Moisture content of microcapsules produced by the different proportions of MD and WPI, and dehydration processes are shown in Table 5. It is noted that the moisture content of the microcapsules is greater when the proportion of MD is higher. MD with DE 19 exhibits some ramifications with hydrophilic groups due to the presence of low molecular weight saccharides [42], contributing to the absorption of water from the environment. Therefore, the values of *T*_g_ of MD−WPI mixtures are reduced with increasing proportions of MD [52]. Higher water activities and lower *T*_g_ (high plasticizing effect of water) may be attributed to MD having DE 19. WPI contains a low number of hydrophilic groups due to less contamination of lactose by membrane processing [46]. It offers lower hygroscopicity of the microcapsules. The moisture contents are lower for microcapsules produced by FD than SD. It may be justified by the fact that microcapsules produced by FD contain high amounts of oil on the surface of the matrix. The hydrophobic nature of oil resists the diffusion of moisture within microcapsules and water activity [15]. Our results are in accordance with the maximum limits specified for dry powders in the food industry, which are between 3% and 4% [51].

#### 3.3.3. Size and Size Distribution (Span) of Microcapsule

The diameter of microcapsules depends on the proportion of MD and WPI, and dehydration process. The values of *D*_43_ and the span of microcapsules, produced by olive oil with different proportions of MD and WPI, and dehydration processes are shown in Table 6. It is noted that the sizes of the microcapsules are higher with a higher proportion of WPI in the emulsion formulation. The microcapsules with a higher proportion of WPI having low *T*_g_ may easily agglomerate during SD [8,53]. It was also noted that the size of the emulsion droplets is higher when the proportion of WPI was greater in the emulsion formulation. Similarly, the span value of microcapsules is greater with a higher proportion of WPI (Table 6). Agglomeration, due to the presence of a higher proportion of WPI, is responsible for the higher values of span. The monodispersed nature of the microcapsules is noted when the proportion of MD is higher in the formulation, and it is associated with a lower span value.

#### 3.3.4. Bulk Density and Tapped Density

The bulk density and tapped density of microcapsules, produced by olive oil with different proportions of MD and WPI, and dehydration processes are reported in Table 7. The bulk density reflects the gap sizes between microcapsules in a static condition, while the tapped density reflects the space between microcapsules, influenced by an external force. Higher bulk density indicates a lower amount of air in the powder void, which can prevent the oxidation of microcapsules. It is noted that both are reduced by increasing the proportion of MD in the microcapsule formulation. This can be justified by the fact that due to the presence of low molecular weight saccharides in MD, microcapsules with a higher proportion of MD were more hygroscopic and adhesive [42,52]. It reduces the space between microcapsules.

#### 3.3.5. Carr Index and Hausner Ratio

The free-flowing characteristics of each microcapsule were evaluated by the Carr index, whereas, the cohesiveness was evaluated using the Hausner ratio [32]. In Table 7, it is noted that the developed microcapsules exhibited poor flowability and high cohesiveness [54], which indicates a high friction among microcapsules [55]. A higher proportion of MD in formulation led to higher values of the Carr index and the Hausner ratio, which signifies reduced flowability of microcapsules. It may be felt that the hydrophilic nature of low molecular weight of carbohydrates in MD having DE 19 might be responsible for the higher interparticle cohesiveness and lower flowability of the microcapsules. The low molecular weight saccharides could absorb moisture from the environment, contributing the agglomeration of microcapsules [32].

#### 3.3.6. Morphology of Microcapsule

**Figure 2:**
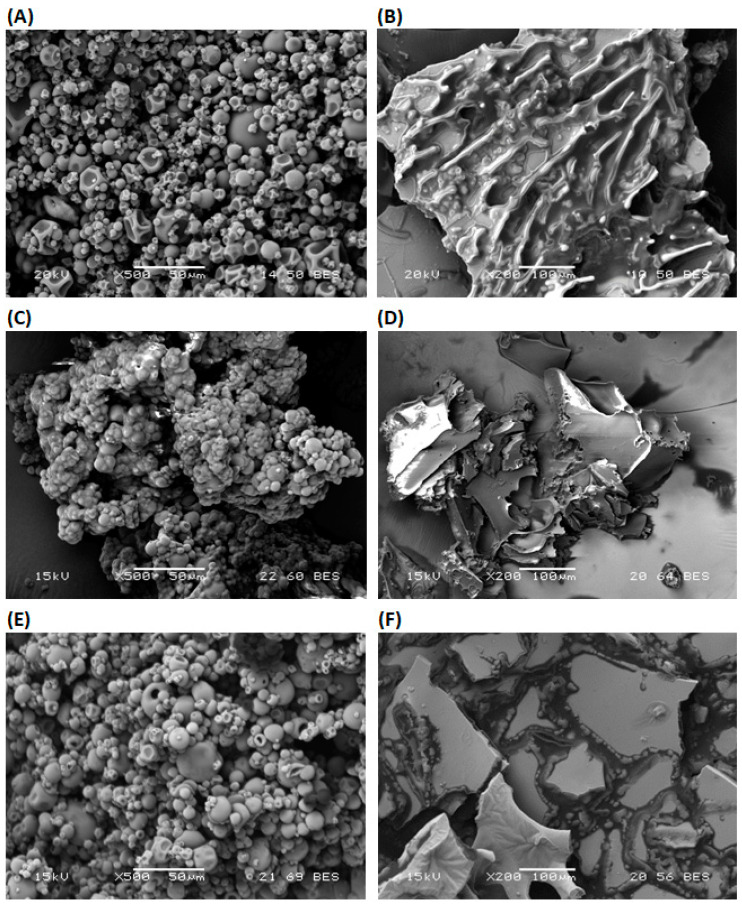
Scanning electron microscopy images of olive oil microcapsule prepared by olive oil with the different proportions of MD and WPI, and dehydration processes (SD, FD); (**A**) 100 WPI SD, (**B**) 100 WPI FD, (**C**) 100 MD SD, (**D**) 100 MD FD, (**E**) 50 MD−50 WPI SD and (**F**) 50 MD−50 WPI FD. MD: Maltodextrin, WPI: Whey protein isolate, SD: Spray-drying, FD: Freeze-drying.

The morphology of microcapsules may be influenced by the characteristics of the emulsion and dehydration process of emulsion. Microcapsules produced by the different proportions of MD and WPI, and dehydration processes are presented in Figure 2.

In general, microcapsules produced by SD are spherical and have a smooth surface without visible pores or cracks. The spherical microcapsules are formed by the melting of MD and WPI in the emulsion due to a high inlet temperature and the nozzle of the spray dryer [56]. Microcapsules produced by FD are flat flakes with an irregular surface. A reduction in the surrounding pressure, crystallization of water in the emulsion and the sublimation of frozen water at a minimal temperature take place during FD [14]. Therefore, dehydration by FD affects the microstructure and the integrity of capsule walls [57].

WPI having a lower value of *T*_g_ may change its form during SD [8]. Therefore, the dent structure of the microcapsules with a smooth surface was produced from the 100 WPI emulsion and SD (Figure 2A). On the other hand, flakes with encapsulated oil were produced from the 100 WPI emulsion and FD. They have a porous irregular surface (Figure 2B). Microcapsules from the 100 MD emulsion and SD or FD have smooth surfaces (Figure 2C,D). MD with the high concentration of lower-molecular-weight saccharides has low *T*_g_. They have a high contribution to water activity and the agglomeration of microcapsules (Figure 2C). Low-molecular-weight saccharides in MD act as a plasticizer during microcapsule formation by SD. Surface morphology was also studied for microcapsules from 50 MD−50 WPI emulsion and SD, because maximum EE was obtained. Microcapsules with spherical smooth surfaces were produced by SD (Figure 2E). For the comparison purpose, microcapsules from 50 MD−50 WPI emulsion and FD were used. It is noted that the flat flakes with some irregularity in surface were produced by FD (Figure 2F).

## 4. Conclusions

Olive oil microcapsules were produced using different proportions of MD with DE 19 and WPI, and subsequent dehydration of emulsion by SD or FD. It was noted that the characteristics of microcapsules, such as EE, moisture content, size and their distribution (span), bulk density, tapped density, flowability, cohesiveness and surface morphology were influenced by the different aspects of emulsion (stability, viscosity, droplet diameter and their distribution) and dehydration processes by SD and FD. The viscosity of the emulsions was increased by higher proportions of WPI in the emulsion, and the stability of the emulsion was reduced. A stable emulsion with a small droplet size was obtained by the higher proportions of MD in the emulsion. The use of MD with WPI provides a unique advantage to produce a stable emulsion as well as the microencapsulation of olive oil. The highest EE (88.61 ± 1.64%) was achieved from the 50 MD−50 WPI emulsion followed by SD. The size of the microcapsules and span values were greater for the higher proportion of WPI having lower values of *T*_g_, which influence the aggregation during SD. Microcapsules with higher proportions of MD contained more moisture due to the presence of low molecular weight saccharides, which are hydrophilic. Bulk density and tapped density of the microcapsules were reduced with an increase in the proportion of MD in the emulsion formulation followed by SD. Furthermore, microcapsules with higher amounts of MD exhibited poor flowability and higher cohesiveness. Microcapsules produced by SD were generally spherical with a smooth surface; however, some dent shaped microcapsules were produced after SD of the 100 WPI emulsion. A smooth surface of the microcapsule was produced due to the presence of MD in formation. Microcapsules produced by FD were flat flakes with an irregular surface due to the sublimation of water and disintegration of the microstructure during FD.

In this investigation, limited numbers of matrix (MD and WPI) and emulsion formulations were used for the microencapsulation of olive oil. Further investigations will be performed to understand the effects of other formulations of emulsions and MD with different DE on the characteristics of microcapsules. Olive oil is enriched with poly-phenolic antioxidants, tocopherols, phytosterols and fatty acids. In the future, investigations will be performed to understand the effects of the dehydration processes on the quality of the encapsulated olive oil and its characteristics at different storage times. Furthermore, the release of encapsulated olive oil from its matrix and the fate of different functional compounds shall be investigated by an in vitro digestion protocol.

## Figures and Tables

**Table 1:** Compositions of emulsions prepared by olive oil with the different proportions of MD and WPI.

	100 WPI	100 MD	50 MD−50 WPI	25 MD−75 WPI	75 MD−25 WPI
MD (g)	0	100	50	25	75
WPI (g)	100	0	50	75	25
Olive oil (g)	40	40	40	40	40
Tween 20 (g)	1	1	1	1	1
DI water (g)	300	300	300	300	300
Olive oil:Matrix	1:2.3	1:2.3	1:2.3	1:2.3	1:2.3

MD: Maltodextrin, WPI: Whey protein isolate.

**Table 2:** Composition of fatty acids and sterols in extra virgin olive oil.

Fatty Acids (g·100 g of oil^−1^)		Sterols (% in Total Sterol)	
Myristic C14:0	0.03 ± 0.001	Cholesterol	0.32 ± 0.008
Palmitic C16:0	13.59 ± 0.01	Brassicasterol	0.03 ± 0.004
*t*-Palmitoleic C16:1	0.31 ± 0.01	24-methylene cholesterol	0.15 ± 0.002
Palmitoleic C16:1n-7	1.15 ± 0.01	Campesterol	2.55 ± 0.02
Stearic C18:0	2.73 ± 0.02	Campestanol	0.17 ± 0.0203
Oleic C18:1 n-9	72.5 ± 0.03	Stigmasterol	1.44 ± 0.023
*c*-Vaccenic C18:1 n-7	0.2 ± 0.01	∆-7-Campesterol	0.11 ± 0.001
*c*-*t* Linoleic C18:2	0.25 ± 0.03	Clerosterol	1.03 ± 0.04
Linoleic C18:2 n-6	7.8 ± 0.04	β-sitosterol	84.51 ± 0.06
Arachidic C20:0	0.45 ± 0.02	Sitostanol	0.65 ± 0.003
Eicosenoic C20:1 n-9	0.25 ± 0.02	∆-5-avenasterol	6.45 ± 0.07
*α*-Linolenic C18:3 n-3	0.75 ± 0.03	∆-5,23-stigmastadienol	0.35 ± 0.003
Eicosadienoic C20:2 n-6	0.12 ± 0.01	∆-5,24-stigmastadienol	0.75 ± 0.0201
Behenic C22:0	0.07 ± 0.008	∆-7-stigmastenol	0.55 ± 0.004
Eicosatrienoic C20:3 n-3	0.21 ± 0.01	∆-7-avenasterol	0.65 ± 0.002
Arachidonic C20:4 n-6	0.48 ± 0.01	Apparent β-sitosterol	92.35 ± 0.32
Lignoceric C24:0	0.08 ± 0.005		

**Table 3:** Characterization of emulsions prepared by olive oil with the different proportions of MD and WPI, expressed by separation (%), droplet diameter (*D*_43_, µm), distribution of droplet size (span) and viscosity (mPA.s).

	Separation (%)	*D*_43_ (µm)	Span (-)	Viscosity (mPa.s)
100 WPI	24.33 ± 0.58 ^b^	4.44 ± 0.04 ^b^	3.12 ± 0.02 ^e^	50.54 ± 0.02 ^e^
100 MD	14.33 ± 0.58 ^a^	2.35 ± 0.03 ^a^	1.52 ± 0.04 ^a^	8.31 ± 0.02 ^a^
25 MD−75 WPI	45.67 ± 0.58 ^e^	5.26 ± 0.04 ^e^	3.04 ± 0.02 ^d^	40.91 ± 0.03 ^d^
50 MD−50 WPI	37.67 ± 0.58 ^d^	5.00 ± 0.01 ^d^	2.69 ± 0.01 ^c^	33.24 ± 0.02 ^c^
75 MD−25 WPI	29.67 ± 0.58 ^c^	4.65 ± 0.01 ^c^	2.53 ± 0.01 ^b^	25.58 ± 0.02 ^b^

MD: Maltodextrin, WPI: Whey protein isolate, results are represented by mean value with standard deviation. In the superscript, a dissimilar alphabet represents the significant difference (Tukey’s post hoc test, *p* < 0.05) between results. According to one-way MANOVA, stability, droplet diameter (*D*_43_), the size distribution of droplets (span) and the viscosity of emulsion are significantly affected by the proportions of MD and WPI (Wilk’s lambda < 0.001, *p* < 0.001). The follow-up one-way ANOVA with Bonferroni’s correction resulted in the significant effect on all four variables (stability, *D*_43_, span and viscosity of emulsion) individually, too (F(4;10) > 1219.61; *p* < 0.001).

**Table 4:** Encapsulation efficiency (EE, %) of microcapsules, produced by olive oil with different proportions of MD and WPI, and dehydration processes.

	EE (%)	
	SD	FD
100 WPI	49.67 ± 1.86 ^Bb^	40.65 ± 0.03 ^Ad^
100 MD	43.49 ± 3.40 ^Bab^	9.93 ± 0.08 ^Aa^
25 MD−75 WPI	54.97 ± 1.10 ^Bb^	17.88 ± 1.20 ^Ab^
50 MD−50 WPI	88.61 ± 1.64 ^Bc^	23.10 ± 0.94 ^Ac^
75 MD−25 WPI	40.62 ± 2.19 ^Ba^	9.92 ± 0.08 ^Aa^

SD: Spray-drying, FD: Freeze-drying, EE: Encapsulation efficiency, MD: Maltodextrin, WPI: Whey protein isolate, Results are represented by mean value with standard deviation. In superscript, a dissimilar alphabet represents the significant difference (Games-Howel’s, *p* < 0.05) between results. Upper case superscripts represent the comparison of dehydration processes within fixed emulsion composition and lower-case superscripts represent the comparison of emulsion compositions within fixed dehydration process. According to two-way MANOVA, the proportions of matrices (MD and WPI) and the dehydration processes, and their interactions had an overall significant contribution on EE of olive oil microcapsule (Wilk’s lambda < 0.001, *p* < 0.001). The follow-up two-way ANOVA with Bonferroni’s correction resulted in a significant effect of matrices, dehydration processes and the interactions of both two variables (F_matrix_(4;20) > 381.22; F_drying_(1;20) > 3562.37; F_interaction_(4;20) > 177.12; all with *p* < 0.001).

**Table 5:** Moisture content (%) of microcapsules produced by olive oil with different proportions of MD and WPI, and dehydration processes.

	Moisture Content %	
	SD	FD
100 WPI	1.70 ± 0.06 ^Ba^	1.03 ± 0.04 ^Aa^
100 MD	3.07 ± 0.05 ^Bd^	2.04 ± 0.03 ^Ac^
25 MD−75 WPI	2.04 ± 0.04 ^Bb^	1.01 ± 0.02 ^Aa^
50 MD−50 WPI	2.45 ± 0.03 ^Bc^	1.02 ± 0.04 ^Aa^
75 MD−25 WPI	3.06 ± 0.03 ^Bd^	1.31 ± 0.03 ^Ab^

SD: Spray-drying, FD: Freeze-drying, EE: Encapsulation efficiency, MD: Maltodextrin, WPI: Whey protein isolate, Results are represented by mean value with standard deviation. In superscript, a dissimilar alphabet represents the significant difference (Games-Howel’s, *p* < 0.05) between results. Upper case superscripts represent the comparison of dehydration processes within fixed emulsion composition and lower-case superscripts represent the comparison of emulsion compositions within fixed dehydration process. According to two-way MANOVA, the proportions of matrices (MD and WPI) and the dehydration processes, and their interactions had an overall significant contribution on moisture content of olive oil microcapsule (Wilk’s lambda < 0.001, *p* < 0.001). The follow-up two-way ANOVA with Bonferroni’s correction resulted in a significant effect of matrices, dehydration processes and the interactions of both two variables (F_matrix_(4;20) > 381.22; F_drying_(1;20) > 3562.37; F_interaction_(4;20) > 177.12; all with *p* < 0.001).

**Table 6:** Particle diameter (*D*_43_, µm) and distribution of particle size (span) of microcapsules, produced by olive oil with different proportions of MD and WPI, and dehydration processes.

	*D*_43_ (µm)		Span (-)	
	SD	FD	SD	FD
100 WPI	9.14 ± 0.06 ^d^	ND	4.30 ± 0.03 ^d^	ND
100 MD	4.94 ± 0.09 ^b^	ND	2.88 ± 0.02 ^c^	ND
25 MD−75 WPI	7.41 ± 0.08 ^c^	ND	4.32 ± 0.02 ^d^	ND
50 MD−50 WPI	4.81 ± 0.02 ^b^	ND	2.69 ± 0.01 ^b^	ND
75 MD−25 WPI	4.22 ± 0.02 ^a^	ND	2.27 ± 0.03 ^a^	ND

SD: Spray-drying, FD: Freeze-drying, EE: Encapsulation efficiency, MD: Maltodextrin, WPI: Whey protein isolate, ND: Not determined. Results are represented by mean value with standard deviation. In superscript, a dissimilar alphabet represents the significant difference (Games-Howel’s, *p* < 0.05) between results. Lower-case superscripts represent the comparison of emulsion compositions within fixed dehydration process. According to one-way MANOVA, the proportions of matrices (MD and WPI) have an overall significant effect on particle size (*D*_43_) and the size distribution of particles (span) of olive oil microcapsule if SD was used (Wilk’s lambda < 0.001, *p* < 0.001). That significant effect was also detected for *D*_43_ and span value of particle individually (one-way univariate ANOVA: F(4;10) > 3644.66; *p* < 0.001).

**Table 7:** Bulk density (g·mL^−1^), tapped density (g·mL^−1^), Carr index (%) and Hausner ratio (-) of microcapsules, produced by olive oil with different proportions of MD and WPI, and dehydration processes.

	Bulk Density (g·mL^−1^)		Tapped Density (g·mL^−1^)		Carr Index (%)		Hausner Ratio (-)	
	SD	FD	SD	FD	SD	FD	SD	FD
100 WPI	0.242 ± 0.002 ^d^	ND	0.305 ± 0.002 ^d^	ND	28.861 ± 0.02 ^a^	ND	1.389 ± 0.002 ^a^	ND
100 MD	0.233 ± 0.001 ^ab^	ND	0.295 ± 0.002 ^bc^	ND	31.252 ± 0.01 ^e^	ND	1.435 ± 0.001 ^e^	ND
25 MD−75 WPI	0.238 ± 0.002 ^cd^	ND	0.298 ± 0.001 ^c^	ND	29.452 ± 0.0 ^b^	ND	1.406 ± 0.00 ^b^	ND
50 MD−50 WPI	0.235 ± 0.001 ^bc^	ND	0.293 ± 0.001 ^ab^	ND	29.751 ± 0.02 ^c^	ND	1.415 ± 0.002 ^c^	ND
75 MD−25 WPI	0.229 ± 0.002 ^a^	ND	0.289 ± 0.002 ^a^	ND	30.512 ± 0.0 ^d^	ND	1.427 ± 0.00 ^d^	ND

SD: Spray-drying, FD: Freeze-drying, MD: Maltodextrin, WPI: Whey protein isolate, ND: Not determined. Results are represented by mean value with standard deviation. In superscript, a dissimilar alphabet represents the significant difference (Tukey’s, *p* < 0.05) between results. According to one-way MANOVA, the proportions of matrices (MD and WPI) have an overall significant effect on bulk density (g·mL^−1^), tapped density (g·mL^−1^), Carr index (%) and Hausner ratio (-) if SD was used (Wilk’s lambda < 0.001, *p* < 0.001). The significant effect was also detected for all the dependent variables individually (one-way univariate ANOVA with Bonferroni’s correction: F(4;10) > 30.78; *p* < 0.001).

## Data Availability

The data presented in this study are available on request from the corresponding author.
